# Supplementary material for: Detailed mapping of Bifidobacterium strain transmission from mother to infant via a dual culture-based and metagenomic approach
Source: Nat Commun. 2023 May 25;14:3015. doi: 10.1038/s41467-023-38694-0 (PMC10213049; doi:10.1038/s41467-023-38694-0)
Supplement: Supplementary file 1 — Supplementary Information [file 41467_2023_38694_MOESM1_ESM.pdf]

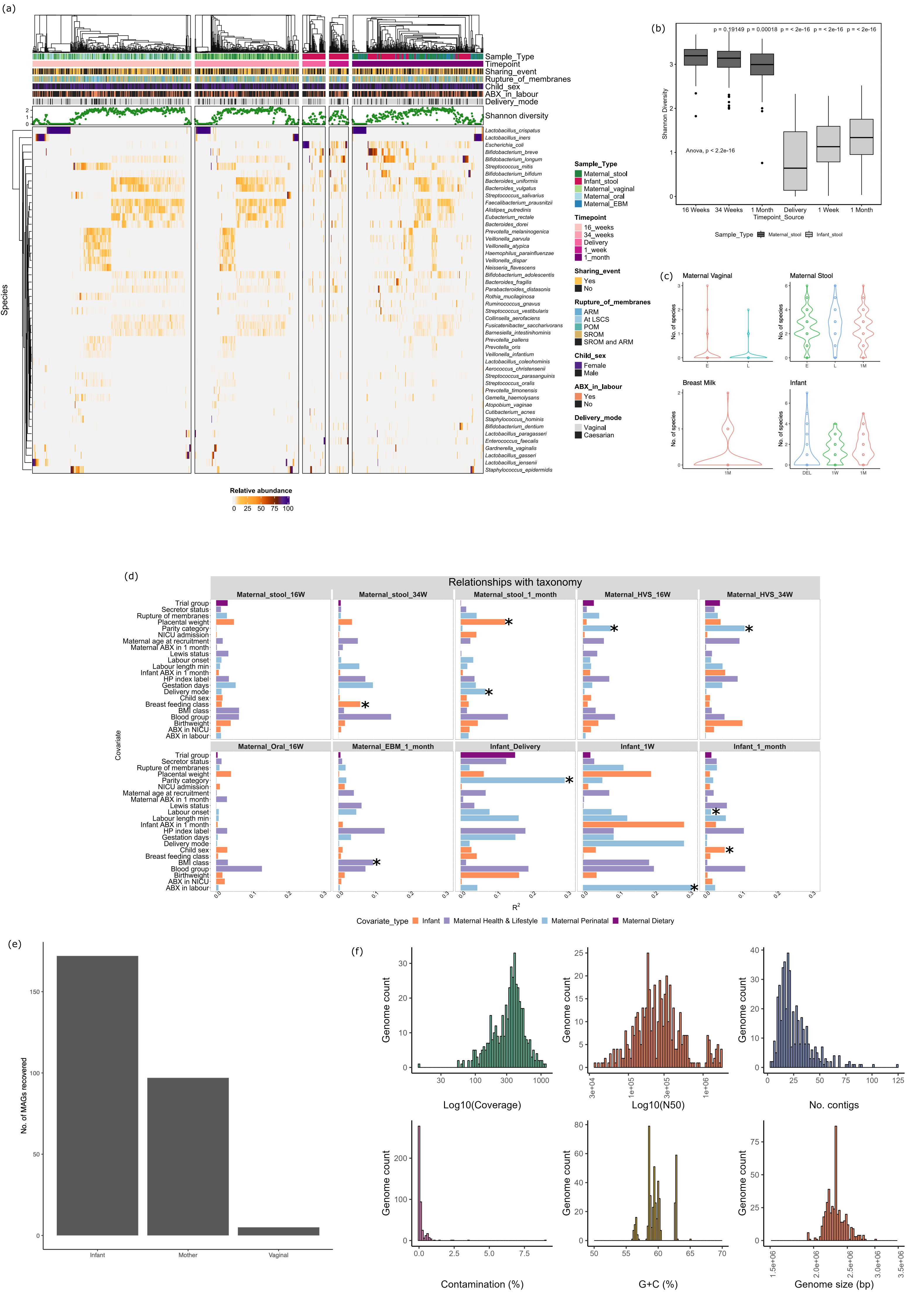

Supplementary Figure 1. a Heatmap representing the top 10 species of each sample type, whereby increased relative abundance for that species goes from yellow to purple on the heat gradient. Each colour bar represents a distinct metadata feature. Samples are clustered by Bray-Curtis, and slices are split by timepoint. **b** Alpha diversity of both maternal (16W n=121, 34W n=129, 1M n=118) and infant (Del n=53, 1W n=45, 1M n=118) stool samples. Boxplots highlight the middle 50% of data with the median relative abundance shown in the central line. The whiskers extend to show the range of values. Asterisks indicate significant differences from two-sided *t*-tests rooted to 16-weeks maternal sample. **c** Number of total *Bifidobacterium* isolated from each sample type split by timepoint. **d** envfit covariate analysis revealing the effect size of 22 different covariates on the microbiota beta diversity of each sample type. Significant associations are denoted with a black asterisk. Infant male/female split is 62/70, respectively. **e** Total number of metagenome assembled genomes (MAGS) recovered from each sample site. **f** Summary statistics for all sequenced *Bifidobacterium* genomes.

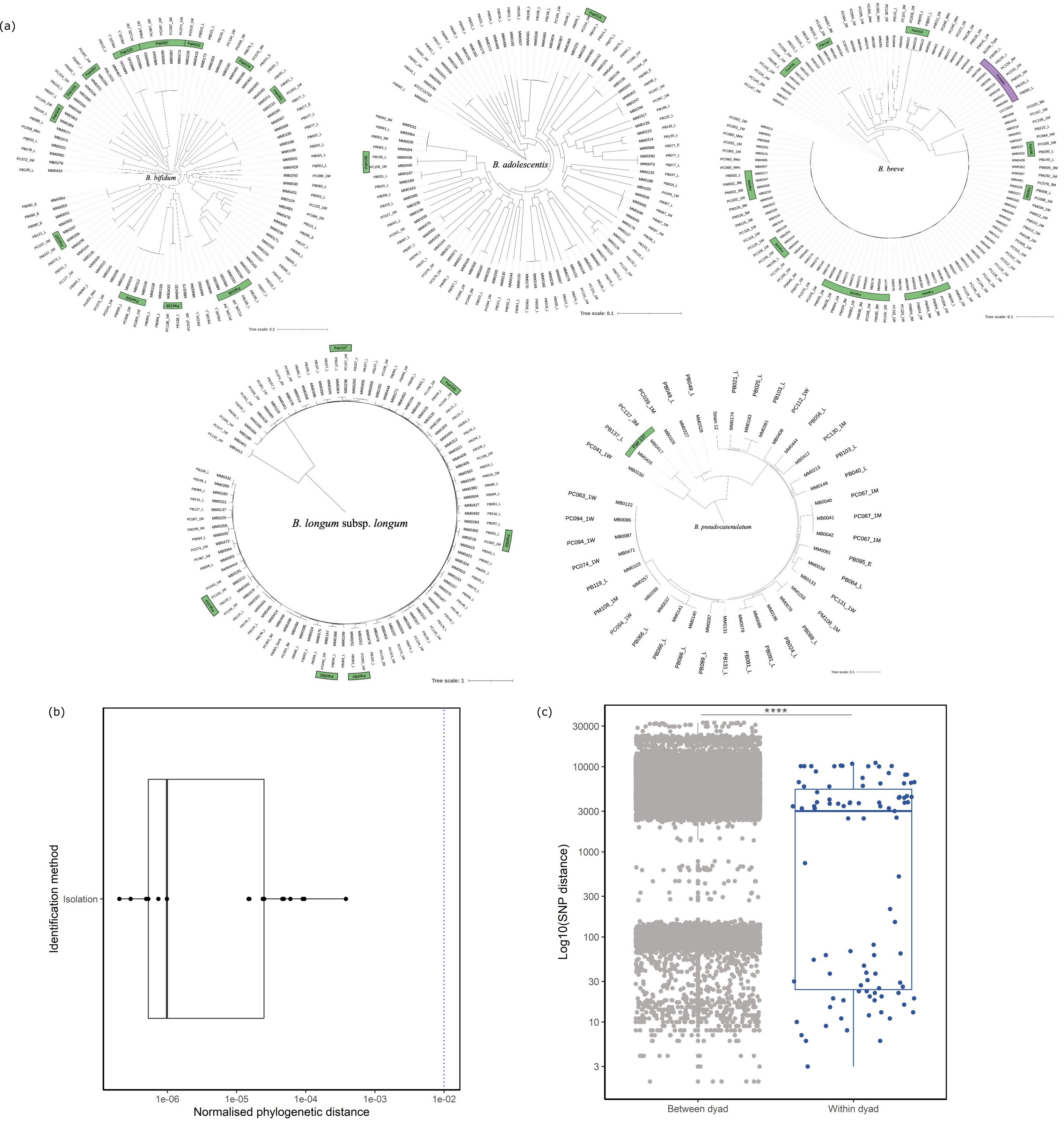

Supplementary Figure 2. a Transmitted *Bifidobacterium* strains identified by strain isolation. Transmitted strains are highlighted by green colour strips in each plot. **b** StrainPhlAn3-determined normalised phylogenetic distance of transmitted trains (n=27) identified by strain isolation. Boxplots highlight the middle 50% of data with the median relative abundance shown in the central line. The whiskers extend to show the range of values. **C** SNP distance of strains between (n=41,129) and within dyads with significant difference (p =1.1x10^{-12}) denoted by asterisk determined by two-sided *t*-test. Boxplots highlight the middle 50% of data with the median relative abundance shown in the central line. The whiskers extend to show the range of values.

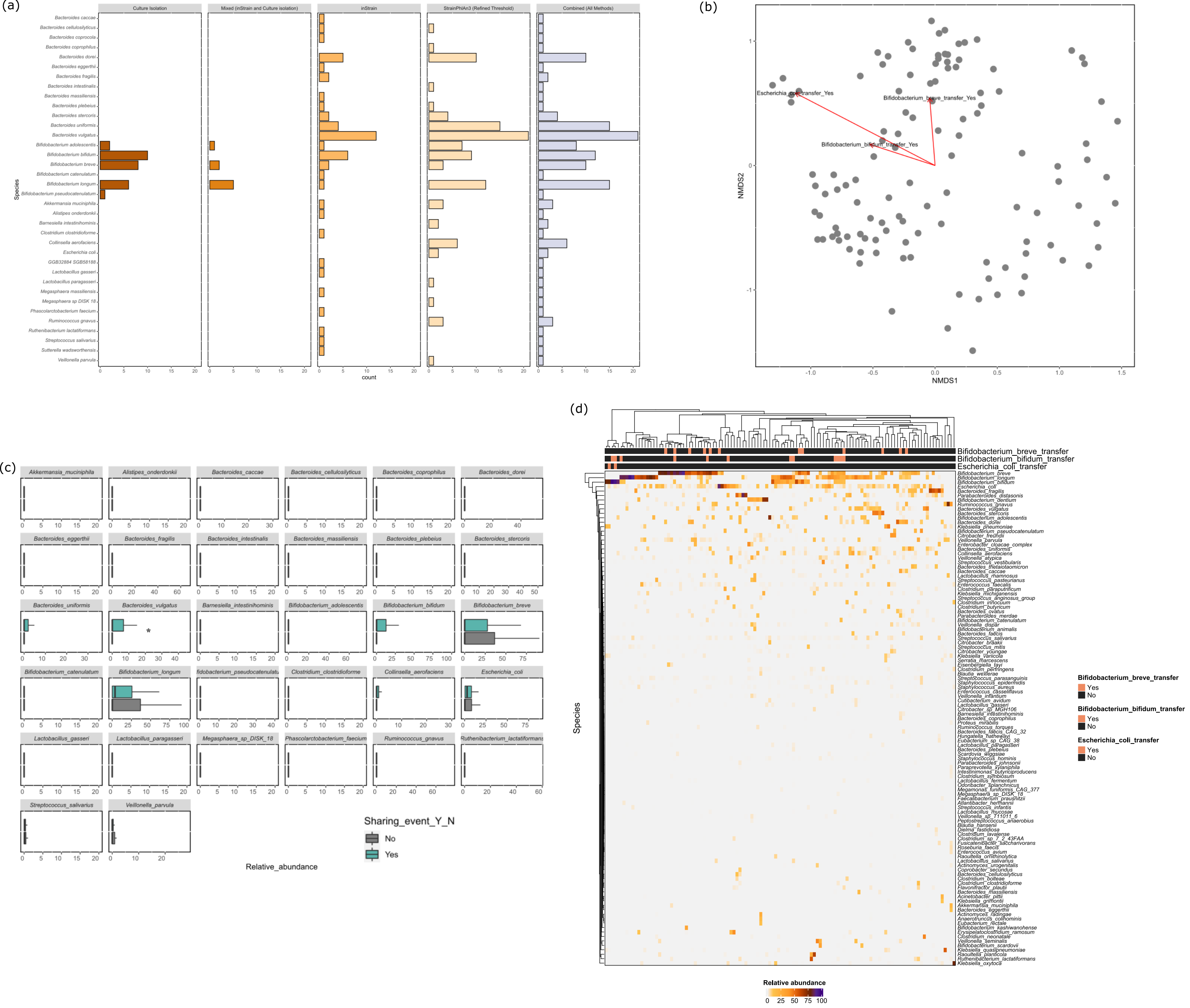

Supplementary Figure 3. a Stratification of all strains transferred by each combination of test method used in this study. **b** nMDS plot of the infant stool microbiome at 1-month, red arrows indicate the direction of effect of significant covariates as determined by envfit. **c** Comparison of the relative abundance within the infant 1-month stool of species of which strains were found to transfer, split by samples that displayed any transfer event. Boxplots highlight the middle 50% of data with the median relative abundance shown in the central line. The whiskers extend to show the range of values. Asterisks indicate significant difference of Bonferroni corrected *p*-value <0.05. **d** Heatmap of all species above 1% relative abundance in at least a single 1-month infant stool sample. Top annotations indicate samples where a transfer of one of the significant covariates from (**b**) are present.

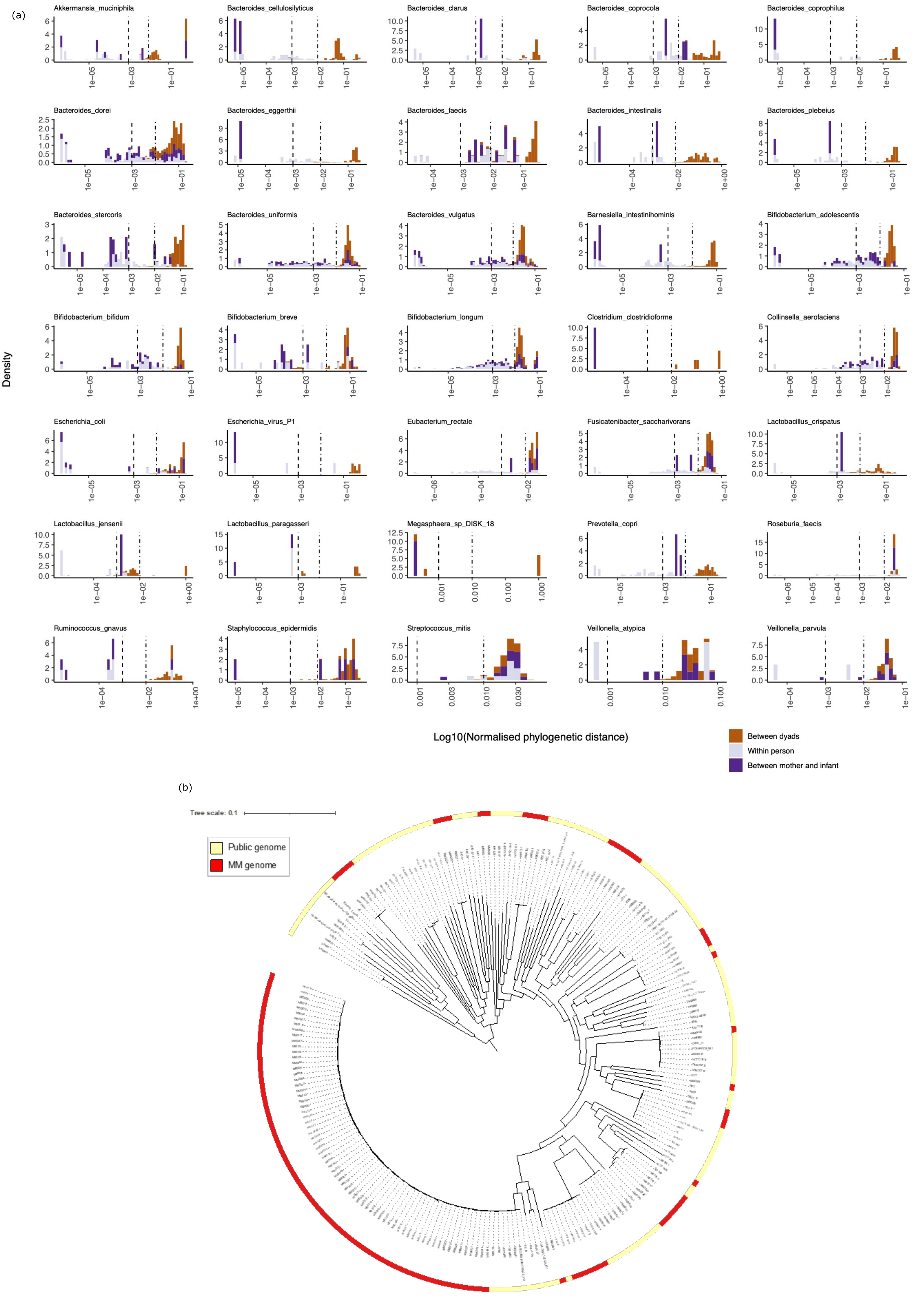

Supplementary Figure 4. a Density plots indicating the number of detected strain sharing as determined by different normalised phylogenetic distance thresholds (horizontal dashed lines). **b** Midpoint rooted phylogenetic tree of alignment of SNP regions of *B. breve* strains from this study and 92 publicly available *B. breve* genomes.
