# Supplementary material for: Detailed mapping of Bifidobacterium strain transmission from mother to infant via a dual culture-based and metagenomic approach
Source: Nat Commun. 2023 May 25;14:3015. doi: 10.1038/s41467-023-38694-0 (PMC10213049; doi:10.1038/s41467-023-38694-0)
Supplement: Supplementary file 3 — Description of Additional Supplementary Files [file 41467_2023_38694_MOESM3_ESM.docx]

File Name: Supplementary Data 1
Description: Summary of dyads

File Name: Supplementary Data 2
Description: Genome sequence information

File Name: Supplementary Data 3
Description: Summary of transmission events with data by method

File Name: Supplementary Data 4
Description: Summary of sharing events

File Name: Supplementary Data 5
Description: Samples with multiple Bifidobacterium genomes

File Name: Supplementary Data 6
Description: Assignment of secretor and Lewis status
