# Supplementary material for: Detailed mapping of Bifidobacterium strain transmission from mother to infant via a dual culture-based and metagenomic approach
Source: Nat Commun. 2023 May 25;14:3015. doi: 10.1038/s41467-023-38694-0 (PMC10213049; doi:10.1038/s41467-023-38694-0)
Supplement: Supplementary file 10 — Reporting Summary [file 41467_2023_38694_MOESM10_ESM.pdf]

nature portfolio

Corresponding author(s): Douwe van Sinderen

Last updated by author(s): Feb 3, 2023

Reporting Summary

Nature Portfolio wishes to improve the reproducibility of the work that we publish. This form provides structure for consistency and transparency in reporting. For further information on Nature Portfolio policies, see our Editorial Policies and the Editorial Policy Checklist.

Statistics

For all statistical analyses, confirm that the following items are present in the figure legend, table legend, main text, or Methods section.

n/a	Confirmed	
☐	☒	The exact sample size (*n*) for each experimental group/condition, given as a discrete number and unit of measurement
☐	☒	A statement on whether measurements were taken from distinct samples or whether the same sample was measured repeatedly
☐	☒	The statistical test(s) used AND whether they are one- or two-sided<br>*Only common tests should be described solely by name; describe more complex techniques in the Methods section.*
☐	☒	A description of all covariates tested
☐	☒	A description of any assumptions or corrections, such as tests of normality and adjustment for multiple comparisons
☐	☒	A full description of the statistical parameters including central tendency (e.g. means) or other basic estimates (e.g. regression coefficient) AND variation (e.g. standard deviation) or associated estimates of uncertainty (e.g. confidence intervals)
☐	☒	For null hypothesis testing, the test statistic (e.g. *F*, *t*, *r*) with confidence intervals, effect sizes, degrees of freedom and *P* value noted<br>*Give P values as exact values whenever suitable.*
☒	☐	For Bayesian analysis, information on the choice of priors and Markov chain Monte Carlo settings
☒	☐	For hierarchical and complex designs, identification of the appropriate level for tests and full reporting of outcomes
☒	☐	Estimates of effect sizes (e.g. Cohen's *d*, Pearson's *r*), indicating how they were calculated

Our web collection on statistics for biologists contains articles on many of the points above.

Software and code

Policy information about availability of computer code

Data collection	Patient information was collected verbally and from medical records. Anonymised data was processed using REDCap v11.1.8 database software.
Data analysis	Statistical analysis and plotting of figures was performed using R, v 4.1.1.<br>Metagenomic analysis of sequence reads was performed with following tools and databses:<br>HumAnN3 v 3.0<br>ChocoPhlAn v 30<br>uniref90 protein database (as bundled with HumAnN3 v 3.0)<br>MetaPhlAn3 v 30<br>StrainPhlAn3 v 3.0<br>MetaSPAdes v 3.14<br>MetaBat2 v 2.12.1<br>checkM v 1.0.18<br><br>Genome analysis of cultured isolated was performed using the following tools and databases:<br>Spades v 3.1<br>Unicycler v 0.4.8<br>canu v 2.2.1<br>circulator v 1.5.5

Prodigal v 2.6.3
BLASTP v 2.6.0
tRNAscan-SE v 1.3.1
Rnammer v 1.2
fastANI v 1.3
trimmomatic v 0.36
snippy v 4.4.5
gubbins v 3.0.0
snp-site v 2.5.1
RAxML v 8.2.12
ape v 5.5

scripts used in this analysis are available on GitHub:
https://github.com/SligoMicrobe/MicrobeMom

For manuscripts utilizing custom algorithms or software that are central to the research but not yet described in published literature, software must be made available to editors and reviewers. We strongly encourage code deposition in a community repository (e.g. GitHub). See the Nature Portfolio guidelines for submitting code & software for further information.

Data

Policy information about availability of data

All manuscripts must include a data availability statement. This statement should provide the following information, where applicable:

- Accession codes, unique identifiers, or web links for publicly available datasets
- A description of any restrictions on data availability
- For clinical datasets or third party data, please ensure that the statement adheres to our policy

A data availability statement is included in the manuscript with all details as requested. All raw sequencing data used in this study is available in the ENA repository under accession number PRJEB48251 (https://www.ebi.ac.uk/ena/browser/view/PRJEB48251).

Human research participants

Policy information about studies involving human research participants and Sex and Gender in Research.

Reporting on sex and gender	The sex of the infant is reported and considered as a covariate in the analysis of microbiome composition.
Population characteristics	A cohort of healthy pregnant women and their subsequent off-spring. Individuals were eligible if they had a body mass index (BMI) between 18·5 and 35 kg/m2, were aged over 18 years, capable of giving informed consent, and had an adequate level of English language to enable study comprehension.
Recruitment	Pregnant individuals were recruited between 15th September 2016 and 12th July 2019. Pregnant individuals were screened for eligibility for the study at their first antenatal visit (ten- and 15-weeks' gestation), through review of their medical chart. Individuals were eligible if they had a body mass index (BMI) between 18.5 and 35 kg/m2, were aged over 18 years, capable of giving informed consent, and had an adequate level of English language to enable study comprehension. Exclusion criteria included history of gestational diabetes (GDM), diabetes mellitus or pre-diabetes, multiple pregnancy, fetal anomaly, previous perinatal death, any medical condition requiring treatment or unwillingness to limit intake of other probiotic food or supplements during the trial.
Ethics oversight	Ethical approval for the study was received from National Maternity Hospital research ethics committee in February 2016 (EC 35.2015). Written informed consent was obtained from all participants and the study was completed in accordance with the Declaration of Helsinki.

Note that full information on the approval of the study protocol must also be provided in the manuscript.

Field-specific reporting

Please select the one below that is the best fit for your research. If you are not sure, read the appropriate sections before making your selection.

[x] Life sciences [ ] Behavioural & social sciences [ ] Ecological, evolutionary & environmental sciences

For a reference copy of the document with all sections, see nature.com/documents/nr-reporting-summary-flat.pdf

Life sciences study design

All studies must disclose on these points even when the disclosure is negative.

Sample size	The analysis in this manuscript was performed on data collected during a randomised controlled trial (MicrobeMom: ISRCTN53023014) and

	power analysis relates to the primary outcome of this investigation. A power analysis determined that 60 participants per group was required to detect a difference in the primary outcome at a significance level of 5%. This was based on an expected colonisation rate in the placebo group of 0% and 15% in the intervention group. The latter number was chosen as the significant difference level in the absence of available literature. A target power of 80% was used, at a type I error rate of 0·05. The final sample size required was 120 infants to provide at least one stool sample in the neonatal period up to 3 months of age.
Data exclusions	Samples that did not have any taxonomic assignment were removed from analysis. In addition, any samples with fewer than 100,000 post-filtering reads were removed. Finally, singleton samples (i.e., maternal samples with no corresponding infant sample) were removed.
Replication	Individual samples were sequenced once, however to control for sequence variation across multiple sequencing runs, three samples were repeated on each run and the microbial composition checked to determine that no significant alterations in composition occurred relative to sequencing runs. All repeat samples shared the same alpha and beta diversity profiles and microbial composition.
Randomization	Randomization was performed for the original study (MicrobeMom: ISRCTN53023014), whereby pregnant women were randomized into either a B. breve supplementation group or a placebo group. This current manuscript is not analyzing the primary outcome of that investigation.
Blinding	All participants and researchers were blinded during the running of the primary trial that generated the data until the primary outcome investigation was complete. As this manuscript deals with a subsequent analysis of the data, not related to the primary outcome, researchers were not blinded for the subsequent analysis.

Reporting for specific materials, systems and methods

We require information from authors about some types of materials, experimental systems and methods used in many studies. Here, indicate whether each material, system or method listed is relevant to your study. If you are not sure if a list item applies to your research, read the appropriate section before selecting a response.

Materials & experimental systems

n/a	Involved in the study
☒	☐ Antibodies
☒	☐ Eukaryotic cell lines
☒	☐ Palaeontology and archaeology
☒	☐ Animals and other organisms
☐	☒ Clinical data
☒	☐ Dual use research of concern

Methods

n/a	Involved in the study
☒	☐ ChIP-seq
☒	☐ Flow cytometry
☒	☐ MRI-based neuroimaging

Clinical data

Policy information about clinical studies

All manuscripts should comply with the ICMJEguidelines for publication of clinical research and a completedCONSORT checklist must be included with all submissions.

Clinical trial registration	ISRCTN53023014
Study protocol	https://www.isrctn.com/ISRCTN53023014?q=ISRCTN53023014&filters=&sort=&offset=1&totalResults=1&page=1&pageSize=10
Data collection	Samples and patient data were collected at the National Maternity Hospital, Dublin, Ireland between 15th September 2016 and 12th July 2019.
Outcomes	Primary outcome measure<br><br>Presence of the supplemented bifidobacterial strain in the infant's stool in the 1st week postpartum, at 1 month postpartum and at 3 months postpartum. To measure this the infant stool will be analysed for the presence of the supplemented bifidobacterial strain.<br><br>Secondary outcome measures<br><br>Differences in the following between the probiotic and placebo groups:<br>1. Maternal lipids<br>2. Maternal insulin resistance as measured by fasting glucose and HOMA-IR<br>3. Maternal C Reactive Peptide<br>Maternal bloods will be taken in early (approx. 16 weeks gestation) and late (approx. 34 weeks gestation) pregnancy and will be analysed for these metabolic parameters.
